# Long Pentraxin 3: Experimental and Clinical Relevance in Cardiovascular Diseases

**DOI:** 10.1155/2013/725102

**Published:** 2013-04-07

**Authors:** Fabrizia Bonacina, Andrea Baragetti, Alberico Luigi Catapano, Giuseppe Danilo Norata

**Affiliations:** ^1^Department of Pharmacological and Biomolecular Sciences, Università Degli Studi di Milano, 20133 Milan, Italy; ^2^Center for the Study of Atherosclerosis, Ospedale Bassini, 20092 Cinisello Balsamo, Italy; ^3^IRCCS Multimedica, 20162 Milan, Italy; ^4^The Blizard Institute, Barts and The London School of Medicine and Dentistry, Queen Mary University, London E1 2AT, UK

## Abstract

Pentraxin 3 (PTX3) is an essential component of the humoral arm of innate immunity and belongs, together with the C-reactive protein (CRP) and other acute phase proteins, to the pentraxins' superfamily: soluble, multifunctional, pattern recognition proteins. Pentraxins share a common C-terminal pentraxin domain, which in the case of PTX3 is coupled to an unrelated long N-terminal domain. PTX3 in humans, like CRP, correlates with surrogate markers of atherosclerosis and is independently associated with the risk of developing vascular events. Studies addressing the potential physiopathological role of CRP in the cardiovascular system were so far inconclusive and have been limited by the fact that the sequence and regulation have not been conserved during evolution between mouse and man. On the contrary, the conservation of sequence, gene organization, and regulation of PTX3 supports the translation of animal model findings in humans. While PTX3 deficiency is associated with increased inflammation, cardiac damage, and atherosclerosis, the overexpression limits carotid restenosis after angioplasty. These observations point to a cardiovascular protective effect of PTX3 potentially associated with the ability of tuning inflammation and favor the hypothesis that the increased levels of PTX3 in subjects with cardiovascular diseases may reflect a protective physiological mechanism, which correlates with the immunoinflammatory response observed in several cardiovascular disorders.

## 1. Introduction

Several inflammatory mediators have been implicated in the pathogenesis of cardiovascular disorders (CVD) [1] and most of them, such as the flogistic molecules CD40 and its soluble ligand, the adhesion molecules ICAM-1, VCAM-1, E-selectin and P-selectin, the peptides NT-proBNP and troponin T, and fibrinogen, are useful as systemic biomarkers for inflammation and tissue damage associated with CVD [2, 3]. However, the acute phase protein which is widely used as a biomarker of cardiovascular and inflammatory disorders is the classical C-reactive protein (CRP), a component of the pentraxin superfamily [4]. Pentraxins are an essential component of the humoral arm of innate immunity and are a superfamily of soluble, multifunctional, pattern recognition proteins characterized by a cyclic multimeric structure [5, 6]. In addition to CRP, the pentraxin superfamily includes the long pentraxin 3 (PTX3), a protein composed by a long characteristic N-terminal domain coupled to the C-terminal pentraxin domain which is emerging as an important player in immunity and inflammation [6]. The aim of this paper will be to discuss the experimental and clinical relevance of PTX3 in cardiovascular diseases.

## 2. The Pentraxin Superfamily

The pentraxin superfamily is characterized by the presence, in the carboxy-terminal region, of the “pentraxin domain”, composed of a conserved 8-amino-acid long sequence (HxCxS/TWxS, where x is any amino acid) [6]. Pentraxins are divided into short and long pentraxins based on the primary structure of the protein: CRP and serum amyloid P-component (SAP) are two well-characterized short pentraxins of about 25-kDa, mainly produced by hepatocytes in response to proinflammatory mediators, such as IL-6 [6, 7]. In humans, plasma levels of CRP are below 3 mg/L under normal conditions but could increase up to 1000 folds in 48 hours during the acute-phase response; this is not true for SAP plasma levels which are relatively stable (30–50 mg/L) even during the acute-phase response [6]. 

PTX3, which is the prototypic long pentraxin, was identified in the early 1990s, as a molecule rapidly induced by IL-1 in endothelial cells (ECs) or by tumor necrosis factor (TNF) in ECs and fibroblast [8, 9]. The protein presents a high degree of conservation from mouse to human (82% identical and 92% conserved amino acids) and is induced in a variety of somatic and innate immunity cells by primary inflammatory stimuli [6]. PTX3 plays a nonredundant role as a soluble pattern recognition receptor for selected pathogens [10] but is also critical for extracellular matrix (ECM) deposition, fertility, and vascular biology [6, 11]. In addition, in humans, PTX3 plasma levels increase during vascular atherosclerosis, inflammation, or damage [12] especially after myocardial infarction and reach the peak much earlier compared to CRP [13]. This prompted the research to investigate whether PTX3 may represent a rapid biomarker for primary local activation of innate immunity and inflammation [11]. 

## 3. PTX3: From Molecular Structure to Physiological Implications

### 3.1. Gene Organization, Protein Structure, and Production

The human PTX3 gene has been localized on chromosome 3 band q25 and is organized in three exons separated by two introns: the first two encode the signal peptide and the N-terminal domain of the protein, respectively, whereas the third exon encodes the C-terminal domain, the pentraxin sequence with high degree of conservation with short pentraxins [8]. The murine gene shows high evolutionary conservation in sequence, gene organization and regulation [14]. Indeed, the proximal promoters of both human and murine PTX3 genes share numerous potential enhancer-binding elements, including activator protein-1 (AP-1), nuclear factor kappa B (NF-*κ*B), and selective promoter factor 1 (SP1). It has been shown that the NF-*κ*B-binding site is essential for the transcriptional response to pro-inflammatory cytokines, as TNF-*α* and interleukin 1*β* (IL-1*β*), whereas AP-1 controls the basal transcription of PTX3 [14, 15].

The mature secreted PTX3 protein consists of a major 45 kD form of monomers, which are assembled to form multimers predominantly of 440 kD apparent molecular mass [6]. The N-glycosylation site in the C-terminal domain [16] affects the binding of PTX3 to a number of ligands and links changes in the glycosylation status with the modulation of the activity [16]. 

PTX3 is induced in several cell types, including ECs, fibroblast, smooth muscle cells (SMCs), adipocytes, mesangial cells, and synoviocytes, following the exposure to several inflammatory signals: IL-1*β*, TNF-*α*, TRL agonists, and microbial components, including lipopolysaccharide (LPS), lipoarabinomannan, outer membrane proteins, peptidoglycan, and oxidized lipids [8, 9, 17–21]. Also in myeloid cells, such as monocytes and dendritic cells (DCs), PTX3 expression is induced by pro-inflammatory stimuli; polymorphonuclear cells (PMNs) instead do not express PTX3 messenger RNA [17] but present the protein stored in specific granules in a ready-to-use form, which is released in response to microorganisms or TLR agonists [22]. In these cells the protein localizes in extracellular traps extruded from activated PMNs (neutrophil extracellular traps) and contribute to the generation of an antimicrobial microenvironment essential to trapping and killing microbes [23, 24]. More recently also anti-inflammatory molecule were shown to modulate PTX3 expression. Glucocorticoid hormones (GCs) induce and enhance the protein expression under inflammatory conditions in fibroblast, but not in myeloid cells [25], while high-density lipoproteins (HDLs), which possess a series of vascular protective activities [26], induce PTX3 expression in endothelial cells [27]. The latter mechanism requires the activation of the PI3K/Akt pathway through G-coupled lysosphingolipid receptors and is mimicked by sphingosine 1 phosphate and other S1P mimetics [27], physiologically present in HDL and responsible for some of the activities linking HDL to the immunoinflammatory response [28].

### 3.2. Multifunctional Properties of PTX3 in Innate Immunity

PTX3 is a key player of the humoral arm of the innate immunity and its physiological functions are associated to the recognition and binding to different ligands, including microbial moieties, complement components, and P-selectin. 

Similarly to short pentraxins, PTX3 recognizes the highly conserved pathogen-associated molecular patterns (PAMPs) expressed by microorganisms [29] and binds a number of bacteria, fungi, and viruses. A specific binding has been observed to conidia of *Aspergillus fumigatus *[10], *Paracoccidioides brasiliensis,* and zymosan [30], to selected gram-positive and gram-negative bacteria [10, 18, 31] and finally to some viral strains, including human and murine cytomegalovirus and influenza virus type A (IVA) [31, 32]. 

Both short pentraxin and PTX3 bind apoptotic cells and facilitate their clearance [33, 34]. Surface bound CRP activates the classical pathway of complement through interaction with C1q, thus leading to cells elimination [35]. Cell-bound PTX3 might favor the clearance of apoptotic cells [34, 36] by enhancing the deposition of both C1q and C3 on cell surface [35]. On the contrary, when in the fluid phase, PTX3 interacts with C1q and dampens the deposition on apoptotic cells and the resulting phagocytosis by DCs and phagocytes [37–40].

In addition to PTX3 C1q recognizes and binds to ficolin-2 and mannose-binding lectin (MBL), thus modulating the classical and the lectin pathways of complement activation [41]. The best described and characterized ligand of PTX3 is the first component of the classical complement system C1q [35, 41]; PTX3 interacts with the globular head of the protein [42] thus resulting in the activation of the classical complement cascade only when C1q is plastic-immobilized, a situation that mimics C1q bound to a microbial surface. On the contrary, when the interaction occurs in the fluid phase, a dose-dependent inhibition of C1q haemolytic activity is observed, suggesting a possible inhibitory effect by competitive blocking of relevant site [35]. A further level of modulation of complement activation by PTX3 is dependent on the glycosylation status of the protein; indeed, deglycosylation or desialylation enhance PTX3 binding to C1q, because of a reduction of the dissociation rate which stabilizes the PTX3/C1q complex [16]. PTX3 interacts with ficolin-2 [43] and the MBL [44], thus promoting also the activity of the lectin-dependent complement pathway and favors complement deposition on *A. fumigatus* conidia and opsonophagocytosis of *Candida albicans* by polymorphonuclear leucocytes [45]. Of note, PTX3 interacts also with Factor H [46] and C4b-binding protein (C4BP) [33, 47], two inhibitors of the complement cascade, thus probably favoring a negative feedback, limiting an exaggerated complement activation. Recently, it has been shown that PTX3 competes with leukocyte PSGL-1 for the interaction with P-selectin through the binding of the N-linked glycosidic moiety in the C-terminal domain of PTX3. This mechanism was shown, in vivo, to inhibit the rolling of leukocytes on P-selectin and to limit P-selectin-dependent inflammation by dampening excessive neutrophil recruitment and extravasation [48].

## 4. PTX3: An Emerging Player in Cardiovascular Diseases

**Figure 1:**
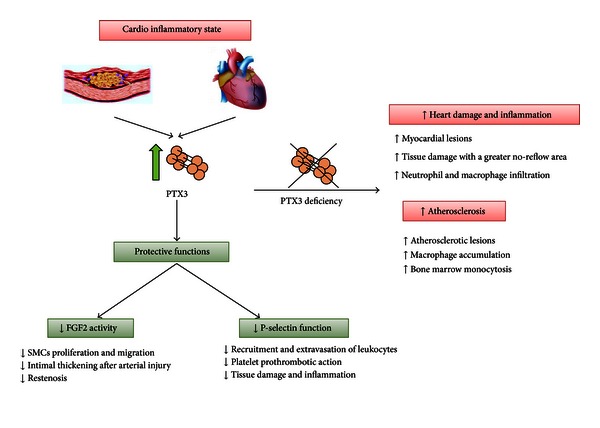
Effects of PTX3 in cardiovascular diseases. PTX3 plasma levels rapidly increase during atherosclerosis and MI in humans and mice. PTX3 could then interact with FGF2 and/or P-selectin and exert cardiovascular protective activities. On the contrary, PTX3 deficiency leads to increased heart damage and inflammation after MI and atherosclerosis.

In the last decade, the presence of PTX3 was detected in the myocardium and in the vasculature under different pathological conditions which was paralleled by the observation of increased plasma PTX3 levels in patients with cardiovascular disorders [12]. These data prompted the research toward the investigation of the role of PTX3 as biomarker, player, or both in the context of cardiovascular disease. So far a role for PTX3 was described in the context of angiogenesis, vascular restenosis, atherosclerosis, and myocardial infarction (Figure 1). 

### 4.1. Role of PTX3 in Angiogenesis and Restenosis

PTX3 dampens fibroblast-growth-factor-2-(FGF2-) dependent ECs proliferation [49] in vitro and this results in the inhibition of angiogenesis, the process of new blood vessel formation [50–52]. Transfection of PTX3 in normal microvascular endothelial cells (MVECs) blunts their angiogenic properties, while PTX3 silencing by small interfering RNA restores the ability of the cells to produce capillaries and promotes angiogenesis [53].

This effect is dependent on the ability of PTX3 to bind with high affinity and selectivity FGF-2 [51, 54] which in turn results in the inhibition of the interaction of FGF2 with tyrosine-kinase receptors (FGFRs) and heparin sulphate proteoglycans (HSPGs) on the surface of ECs and SMCs, thus dampening the generation of the proangiogenic complex HSPG/FGF2/FGFR [20]. 

Interestingly, TSG6, the secreted product of tumor necrosis factor-stimulated gene, reverts the inhibitory effects exerted by PTX3 on FGF2-dependent angiogenesis through competition with FGF2/PTX3 interaction, thus exerting a proangiogenic function [55]. The interaction between TSG-6 and the N-terminal domain of PTX3 plays also a fundamental role in female fertility: TSG-6, the glycosaminoglycan hyaluronan (HA), inter-*α*-trypsin inhibitor (I*α*I), and PTX3 all cooperate in the formation of an ECM around the preovulatory oocyte, the cumulus oophorus complex (COC) matrix [56, 57]. These interactions are essential for the correct organization of the viscoelastic matrix of cumulus oophorus and the lack of PTX3 is associated with female subfertility, as a consequence of cumulus matrix instability [58, 59].

The FGF/FGFR system plays also a crucial role in SMC proliferation, migration, and survival in vitro [60–63] and neointimal thickening after arterial injury in vivo [64, 65]. These processes are critically involved in restenosis, the process of blood vessel narrowing that frequently occurs after percutaneous transluminal coronary angioplasty of atherosclerotic arteries. The possibility that PTX3, by interacting with FGF2, could inhibit FGF2-dependent SMCs activation and intimal thickening after carotid injury is indeed intriguing. Experimental data confirmed this hypothesis and the overexpression of PTX3 in vivo or exogenously added PTX3 in vitro resulted in the inhibition of FGF2-dependent SMCs proliferation [54]. This effect was associated with the suppression of the mitogenic and chemotactic activities exerted by endogenous FGF2 on these, sequestering the growth factor in an inactive form [54]. 

These observations suggest that PTX3 could act as an “FGF2 decoy” and may represent a potent inhibitor of the autocrine and paracrine stimulations exerted by FGF2 on SMCs and point to a novel therapeutic role of PTX3 in the treatment of restenosis after angioplasty [45].

### 4.2. PTX3 and Atherosclerosis

Immunohistochemical staining of advanced atherosclerotic lesions revealed a strong expression of PTX3 on the surface of lumen as well as within the atherosclerotic plaque in animal models and in humans [66, 67]. Cholesterol accumulation in the intima of the vessels, the major pathological feature of atherosclerosis, is associated with the induction of an immune-inflammatory response resulting in the recruitment of monocyte/macrophages, PMNs and in the activation of ECs, which are all able to produce PTX3 in response to inflammatory stimuli normally associated with atherogenesis. IL-1 and TNF*α* are both expressed in advance atherosclerotic lesions [68–70] and these molecules are major candidates for regulating PTX3 expression [67]. Moreover, it has been demonstrated that the disruption of the internal layers of the vessel, after coronary stenting in patients with severe atherosclerosis, causes the increase of PTX3 plasma levels already after 15 minutes [71]. Furthermore, neutrophils and monocytes/macrophages could contribute to the large amount of PTX3 observed in arterial thrombi and aortic tissue in patients with different degrees of atherosclerosis with acute myocardial infarction [72]. 

Oxidized LDL (but not native LDL) increases PTX3 mRNA expression in vascular SMCs (VSMCs), an effect dependent on NF-*κ*B activation [21]. In turn, PTX3 could favor the clearance of lipid-loaded macrophages and VSMCs apoptotic cells mediating their removal by mature DCs [21]. A similar effect could rely on the atheroprotective activity of HDL that was shown to induce PTX3 expression [28] and potentially promote the same mechanism [73].

The potential role of PTX3 in atherosclerosis was recently addressed in PTX3/apolipoprotein E (ApoE) double knockout mice [66]. Normally mice are poorly susceptible to atherosclerosis [74]; therefore, the effects of the deficiency or the overexpressions of a protein have to be tested in mice with a background susceptible to atherosclerosis. For this reason, specific transgenic mice were generated and among them, mice lacking apoE or LDL-receptor are widely used [74–76]. 

PTX3 KO/Apo E KO mice developed larger atherosclerotic lesions compared to Apo E KO, an observation that was coupled to increased macrophage accumulation, increased bone marrow monocytosis, and, interestingly, increased expression of adhesion molecules, cytokines, and chemokines in the vascular wall. These findings suggest an increased immune-inflammatory response in PTX3 KO/Apo E KO mice compared to Apo E KO animals [66]. 

PTX3 expression, similar to that of CRP, is increased also in coronary plaques of patients with unstable angina pectoris (UAP) compared to those with stable angina (SAP). Nevertheless, PTX3 and CRP distribution is different; PTX3 expression is increased in complicated plaque compared to fibroatheroma, while the opposite is true for CRP; moreover abundant PTX3 was detected in intraplaque hemorrhage, while CRP staining was more intense in lipid rich plaque [77]. Finally PTX3 is highly expressed in CD163-positive areas which normally marks anti-inflammatory M2 macrophages [77].

In summary, PTX3 is produced following stimulation of macrophages, SMCs, and ECs with LPS, IL-1, TNF, or oxLDL and is able to induce tissue factor [78, 79]. Functional data clearly point to a cardiovascular protective effect [12] which is associated with the ability to dampen ischemic heart disease and atherosclerosis. 

### 4.3. PTX3 in Ischemic Heart Disease

Plasma concentration of PTX3 rapidly rises in the early phase after ischemic heart disorders in humans and animal models [13, 67, 80, 81] suggesting the possibility that PTX3 could also play a role in myocardial infarction. To this aim coronary artery ligation and reperfusion was performed in PTX3-deficient mice [81].

Following myocardial infarction, increased PTX3 mRNA and protein expression was observed in the ischemic area of the heart. PTX3 KO mice had greater myocardial lesions, an increased tissue damage with a greater no-reflow area, increased neutrophil and macrophage infiltration, decreased number of capillaries, and increased number of apoptotic cardiomyocytes, a phenotype reversed by the use of exogenous PTX3 [81]. The possibility that PTX3 could dampen an excessive complement system activation is supported by the observation that C3 deposition is increased in infarct areas of PTX3 KO mice [81]. Of note, in the intestinal ischemia/reperfusion model, obtained by the total occlusion of the superior mesenteric artery, Souza and colleagues showed that PTX3 overexpression is associated with increased cytokines production, tissue inflammation, and a reduced survival [82]. This finding was later confirmed in PTX3 KO mice which showed an opposite profile [83].

These works clearly highlight different roles played by PTX3 in mediating reperfusion injury in the heart, where its function is protective and localized, rather than the intestine, where PTX3 has a harmful role [83] and could also indicate that the molecule may have different functions in different settings or temporal windows of vascular pathology.

More recently, the identification of P-selectin as a target protein of PTX3 [48] raised the possibility that this mechanism could be relevant in disorders where the excessive neutrophil recruitment to activated endothelium and collateral damages associated to leukocyte activation such as acute coronary syndromes could play a role [84]. 

Maugeri and colleagues identified PTX3 stored in neutrophils' secondary granules as an attractive candidate that leads to the increased plasma levels of the protein after early AMI [85]. The depletion of neutrophil intracellular PTX3 was also associated with increased platelets-neutrophil aggregates and with the binding between PTX3 and activated platelets, which dampens their inflammatory potential and prothrombotic action [85]. Future studies should investigate whether PTX3 might exert a protective role in AMI by dampening the neutrophil-activation loop during atherothrombosis. 

## 5. PTX3: A Clinical Biomarker of Cardiovascular Inflammation

**Figure 2:**
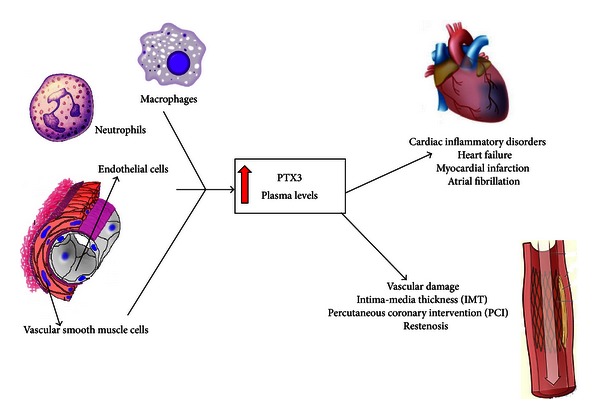
PTX3 as a marker of cardiovascular inflammation. A variety of cell types produce PTX3 in response to pro- and anti-inflammatory signals. Increased PTX3 levels are useful as a marker of different cardiovascular diseases including (a) cardiac inflammatory disorders, such as HF, MI, and atrial fibrillation, and (b) vascular damage, as IMT, PCI, and restenosis.

Following myocardial infarction (MI), PTX3 plasma levels peak within 7.5 hours as compared to CRP which peaks around 50 hours [13]. In MI patients, PTX3 but not CRP, after adjustment for major risk factors and other acute phase proteins, independently predicted 3-month mortality [80]. These data, suggesting PTX3 as a strong prognostic marker of CVD death, paved the road for several studies addressing its role as a biomarker of cardiovascular inflammation (Figure 2) independently of other acute phase proteins such as CRP. 

### 5.1. PTX3 in Subclinical Atherosclerosis and Peripheral Vascular Diseases

Ultrasound detection and quantification of the common carotid artery wall thickness (intima-media thickness, IMT) (CCA-IMT) is considered a surrogate marker of subclinical atherosclerosis [86].

In the Bruneck study, PTX3 plasma levels, although not correlated with CCA-IMT, were higher in individuals with atherosclerotic plaques and prevalent vascular diseases [87]. 

Also in patients with metabolic syndrome, PTX3 levels were directly correlated with CCA-IMT [88]; this correlation, however, was no longer significant after adjustment for HDL-C levels. As not only PTX3 but also HDL sense changes in the immune response [89], the possibility that, in patients with metabolic syndrome, changes in PTX3 or HDL-C levels could both reflect similar alterations in the immunoinflammatory profile should not be excluded. The analysis of the predictive value of PTX3 levels on IMT progression, in addition to the association with IMT, is warranted to support the relevance of PTX3 as a marker of preclinical atherosclerosis.

PTX3 levels, together with proteinuria, were also shown to be independently associated with endothelial dysfunction in end-stage renal disease patients [90]; this suggests the possibility that PTX3 could also represent a biomarker of peripheral vascular damage. Studies investigating PTX3 levels in relation to arterial stiffness, peripheral artery disease (PAD), or pulse wave velocity (PWV) are warranted to clarify this issue. 

### 5.2. PTX3 in Acute Coronary Syndromes

PTX3 is present in the intact myocardium, increases in the blood of patients with acute myocardial infarction, and represent an early indicator of myocytes irreversible injury in ischemic cardiomyopathy [13]. 

In a large cohort of patients with myocardial infarction, with ST elevation, PTX3 but not the liver-derived short pentraxin CRP or other cardiac biomarkers (NT-proBNP, TnT, CK) predicted 3-month mortality after adjustment for major risk factors and other acute-phase prognostic markers [80]. Of note, ECG-documented postinfarction angina, re-infarction, or induced ischemia after the provocative test was not affected by this marker. A similar observation was confirmed in the Cardiovascular Health Study where PTX3 was showed to be independently associated with increased risk of all-cause death and cardiovascular-disease-(CVD-) related mortality (also after adjusting for all major cardio-metabolic risk factors) [91]. Further studies confirmed that PTX3 represents a more specific marker for acute coronary syndrome (ACS) compared to neutrophil activating peptide-2 (NAP-2) and cardiac troponin I (cTnI) in patients with unstable angina pectoris, NSTEMI, and STEMI within the first six hours of the onset of chest pain [92]; furthermore its coronary sinus plasma levels positively correlate with the Gensini score and negatively with multidetector-row computed tomography plaque density (while hsCRP did not) [93]. These findings support the concept of PTX3 as a very sensitive marker of plaque inflammation and vulnerability and of the prognosis of the coronary disease. 

Patients with unstable angina pectoris and those who undergo percutaneous coronary intervention generally present PTX3 levels three times higher than the reference range [94]. Coronary stenting enhances circulating PTX3 levels in association with an inflammatory response and the levels are positively correlated with the increase in activated Mac-1 on the surface of neutrophils at 48 h in the coronary sinus [71]. These data suggest that PTX3 may be a useful marker for evaluation of inflammatory reaction and neointimal thickening after coronary vascular injury and also myocardial infarction.

### 5.3. PTX3 in Heart Failure

The role of inflammation in the progression of chronic heart failure (HF) is debated. Among the different markers of inflammation tested PTX3 was consistently associated with outcomes in HF patients [95]. In two large independent clinical trials (CORONA and GISSI-HF), baseline elevated PTX3 was associated with a higher risk of all-cause mortality cardiovascular mortality or hospitalization for worsening HF. Three-month changes in PTX3 were associated with fatal events after adjustment for hsCRP or NT-proBNP [95].

PTX3 levels were positively associated with the severity of dilatative cardiomyopathy and increased risk of HF [96]. Furthermore high PTX3 plasma levels (but not CRP, interleukin-6, or TNF-*α*) were associated with an echocardiographic measure of left ventricular dysfunction (*E*/*e*′ ratio) in controls without HF and in patients with HF and normal or reduced ejection fraction [97]. PTX3 levels were significantly elevated in patients with HF but normal ejection fraction (HFNEF) and the protein was observed in the coronary circulation in patients with left ventricular diastolic dysfunction (LVDD) [97] indicating that PTX3, but not high-sensitivity CRP, is an independent inflammatory marker correlated with the presence of LVDD and HFNEF. 

The clinical application of assessing blood PTX3 for predicting HF development is debated [98] and studies addressing how PTX3 would compare with established HF markers, such as B-type natriuretic peptide, are warranted.

## 6. Conclusions

PTX3 has emerged as a key acute-phase protein associated with inflammation in cardiovascular disorders, including heart failure, atherosclerosis, acute coronary syndromes, and peripheral vascular diseases. More importantly the predictive value of PTX3 appears to be independent of other risk factors including other markers of the same superfamily such as CRP. The rapid increase of PTX3 plasma levels during cardiovascular events as a consequence of rapid synthesis by various cell types supports the idea that PTX3 could be an early indicator of the activation of both immune and inflammatory responses. 

Data from animal models suggest that this increase might reflect a feedback mechanism involved in dampening an excessive inflammation rather than only the consequence of the activation of the immunoinflammatory system. The ability of PTX3 to control infections and subsequent inflammation will fit with this hypothesis [10, 48]. 

In conclusion, available data indicate PTX3 as a potential target for therapy. While rosuvastatin was shown to increase PTX3 levels in patients with HF this activity was not associated with a beneficial effect [95]. On the contrary pitavastatin treatment was associated with reduced PTX3 levels [99]. The pharmacological relevance of targeting PTX3 in relation to clinical benefits would represent therefore a main subject of investigation for further studies.
